# Health Tract, No. 176

**Published:** 1863-11

**Authors:** 


HEALTH TRACT, No. 176.

### POSTURE IN WORSHIP.

Of all the lazy folks in creation, old-school Presbyterians take the lead in reference to the manner in which they conduct religious worship on the Sabbath-day. Every principle of physiology and common-sense is subverted; every instinct of propriety, respect, reverence, and devotion are all sacrificed to the Moloch of personal idleness and ease. The people go in, squat down on benches, and sit and sit and sit for two mortal hours, neither kneeling nor standing until two or three minutes previous and preparatory toward taking their hats and marching out. Some denominations have the decency to kneel in prayer, which seems very appropriate and becoming; the Presbyterian leans forward, spreads out his elbows along the pew-back for about a yard, leans his forehead on his hands and goes to sleep, becomes semi-comatose, or lays plans for next day. Some of them, the women, doubtless are devout as far as persons can be who can scarcely keep their eyes open. Does it not defy criticism, that keeping one position for nearly two hours predisposes to sleep, which is further cherished and invited by leaning forward, as just described, and closing the eyes. Episcopalians are called formal by some, and ceremonious, by their frequent change of position in sitting, standing, and kneeling; others derisively speak of it as "bobbing up and down all the time," so that a stranger can't tell what's what, as sometimes they sit when they sing, at others stand when they sing; now the minister recites, and they stand; again he recites, and they sit; a third time, and they lean forward; sometimes he says, "Amen!" and they lean on, take no notice of it; at another time he says, "Amen!" and "as you were" seems to be the order of the day. We never fail to get mixed up entirely when we go to hear the Episcopals preach; nor have we any chance of going to sleep. Who ever sits squat down two hours at a stretch at home, abroad, anywhere on the face of the earth, except a Presbyterian at public worship? It is the more irrational, in proportion as the worshiper is a laboring man, or is actively engaged in business during the week, for the blood will tend to stagnation from the long one position, the body becomes uneasy and cries out for change, as is evidenced plainly enough by the incessant wriggling about in the pew; while the brain is oppressed by the stagnating blood, and the mind works sluggishly and sleepily. The good old-fashioned Methodist plan is the best, the most rational, devout, and becoming; to sit when they listen to man; to kneel when they address the Great I Am; to stand when they praise before the Saviour of all. But homely old Methodism is getting out of date now; it isn't decorous in these times to "shout aloud" and show that the worshiper is a wide-awake Christian, a living man; they don't sing in these times as if they would split their throats open with the gushing unction of their songs, but they are getting to be put in strait-jackets like other people, with "steepelows" to their churches, and doors to their pews, as if to keep out the uncircumcised and the stranger; while their foretime soul-singing has dwindled down to a prim squeak, like a penny whistle that had the croup. What would good old John Wesley say, if he could be resurrected?

